# Somatotopical feedback versus non-somatotopical feedback for phantom digit sensation on amputees using electrotactile stimulation

**DOI:** 10.1186/s12984-015-0037-1

**Published:** 2015-05-02

**Authors:** Dingguo Zhang, Heng Xu, Peter B Shull, Jianrong Liu, Xiangyang Zhu

**Affiliations:** State Key Laboratory of Mechanical System and Vibration, Institute of Robotics, School of Mechanical Engineering at Shanghai Jiao Tong University, Shanghai, China; Department of Neurology, Ruijin Hospital, Shanghai Jiao Tong University, School of Medicine, Shanghai, China

**Keywords:** Electrotactile stimulation, Phantom digit somatotopy, Somatotopical feedback, Non-Somatotopical feedback, Amputees

## Abstract

**Background:**

Transcutaneous electrical stimulation can provide amputees with tactile feedback for better manipulating an advanced prosthesis. In general, there are two ways to transfer the stimulus to the skin: somatotopical feedback (SF) that stimulates the phantom digit somatotopy on the stump and non-somatotopical feedback (NF) that stimulates other positions on the human body.

**Methods:**

To investigate the difference between SF and NF, electrotactile experiments were conducted on seven amputees. Electrical stimulation was applied via a complete phantom map to the residual limb (SF) and to the upper arm (NF) separately. The behavior results of discrimination accuracy and response time were used to examine: 1) performance differences between SF and NF for discriminating position, type and strength of tactile feedback; 2) performance differences between SF and NF for one channel (1C), three channels (3C), and five channels (5C). NASA-TLX standardized testing was used to determine differences in mental workload between SF and NF.

**Results:**

The grand-averaged discrimination accuracy for SF was 6% higher than NF, and the average response time for SF was 600 ms faster than NF. SF is better than NF for position, type, strength, and the overall modality regarding both accuracy and response time except for 1C modality (*p*<0.001). Among the six modalities of stimulation channels, performance of 1C/SF was the best, which was similar to that of 1C/NF and 3C/SF; performance of 3C/NF was similar to that of 5C/SF; performance of 5C/NF was the worst. NASA-TLX scores indicated that mental workload increased as the number of stimulation channels increased.

**Conclusions:**

We quantified the difference between SF and NF, and the influence of different number of stimulation channels. SF was better than NF in general, but the practical issues such as the limited area of stumps could constrain the use of SF. We found that more channels increased the amount and richness of information to the amputee while fewer channels resulted in higher performance, and thus the 3C/SF modality was a good compromise. Based on this study, we provide possible solutions to the practical problems involving the implementation of tactile feedback for amputees. These results are expected to promote the application of SF and NF tactile feedback for amputees in the future.

## Background

Currently, vision is the predominant feedback for upper limb amputees to control prosthetic hands, while tactile feedback is conspicuously missing in practical applications [1-3]. This results in high mental workload for amputees and impairs the ability of amputees to control advanced prosthetic hands of high dexterity [4]. Researchers have been investigating the use of artificial stimulation for providing amputees with tactile feedback information, in order to achieve more dexterous prosthetic control while reducing the mental load of users. Electrotactile stimulation can stimulate afferent receptors in the skin, resulting in perceived sensations like vibration, pressure, and slipping [5-7]. Besides prosthesis, electrotactile stimulation has been widely used in other fields such as tactile sensory substitution, teleoperation, robotics, and virtual reality [8-10].

Artificial tactile feedback can be delivered to amputees in two ways: somatotopical feedback (SF) that stimulates the phantom digit somatotopy on the stump [11-16] and non-somatotopical feedback (NF) that stimulates other positions on the body [17-22]. Most researchers choose NF to deliver tactile sensory information and stimulate some ordinary sites such as forearm [18,21,23-25], lower back [20,26], or other places on the body [17]. Though some researchers stimulate the stump to deliver tactile feedback, they typically choose positions that have two-point discrimination, rather than positions that have certain phantom digit somatotopy [12,14]. Recent research has shown that some forearm amputees have phantom hand sensations causing them to feel that their fingers or other parts of the missing hand are being touched when some areas of their stumps are touched [27,28]. However, there are few studies that utilize SF for closed-loop prosthetic control [13]. Thus, differences between SF and NF have not been investigated. Therefore, it is important to quantify the difference between NF and SF based on a thorough comparative study. Further, many researchers have analyzed the tactile sensory ability of subjects based on five channels for five fingers [12,14,16,18,19,21,23,24,29]. Due to practical concerns, a balance between rich feedback information and high performance is needed. It means that some channels need to be removed to achieve better discrimination performance of tactile feedback. In addition, a majority of studies have investigated the behavior of able-bodied subjects for providing amputees with tactile feedback [19,20,24,25]. However, differences between intact-limbed persons and amputees in perceiving, interpreting and utilizing artificial tactile feedback are significant. Finally, current methods typically only examine discrimination accuracy on different sensations, and rarely analyze the response time and the user’s mental workload [2].

To comprehensively compare SF and NF on transradial amputees, we investigate the following three aspects based on electrotactile stimulation. First, NF requires forearm amputees to “learn” the new mapping between finger tactile sensation and unrelated skin areas (e.g. upper arm), while SF exploits the remaining mapping of amputees. The key question is what the difference is between the two mental tasks in the context of prosthetic control. To answer this question, this study quantitatively investigates the different effects between SF and NF in discriminating multi-position, multi-type and multi-strength electrotactile sensations on seven transradial amputees. Second, this study aims to analyze the performance differences between SF and NF under different numbers of stimulation channels. Third, we investigate the mental workload of the amputees using SF and NF based on the NASA-TLX self-assessment questionnaire.

In this work, we evaluate the performance of multi-position, multi-type, and multi-strength electrotactile stimulation on SF and NF with different numbers of channels, and provide suggestions and guidelines for implementing artificial electrotactile feedback for amputees.

## Material and methods

### Participants

Seven transradial amputees participated in the experiment voluntarily, and all had five phantom digits on the residual limbs (Table 1). Every subject was informed of the procedure of the experiment and signed a written informed consent. The study was approved by the local Ethics Committee of Shanghai Jiao Tong University where the experiments took place.

**Table 1:** **Subject demographics**

**Subject**	**Handedness**	**Lower arm**	**Cause of**	**Time after**	**Daily prosthesis**		
**(gender, age)**		**stump length**	**amputation**	**amputation**	**usage/type**		
		**(cm)**		**(years)**		**(VAS)**	
Subject 1(M,72)	R	R. mid third (15)	Traumatic	34	Half day, myoelectric	Yes (5)	
Subject 2(M,35)	R	R. lower third (23)	Tumor	7	All day, cosmetic	Yes (4)	
Subject 3(F,56)	R	R. upper third (8)	Traumatic	31	All day, cosmetic	Yes (4)	
Subject 4(M,60)	R	L. mid third (16)	Traumatic	7	Half day, cosmetic	Yes (4)	
Subject 5(F,57)	R	L. mid third (17)	Traumatic	30	Half day, cosmetic	Yes (5)	
Subject 6(F,41)	R	R. mid third (19)	Traumatic	2	All day, myoelectric	Yes (2-3)	
Subject 7(F,50)	R	L. upper third (10)	Traumatic	25	Half day, myoelectric	Yes (1)	

### Devices

**Figure 1:**
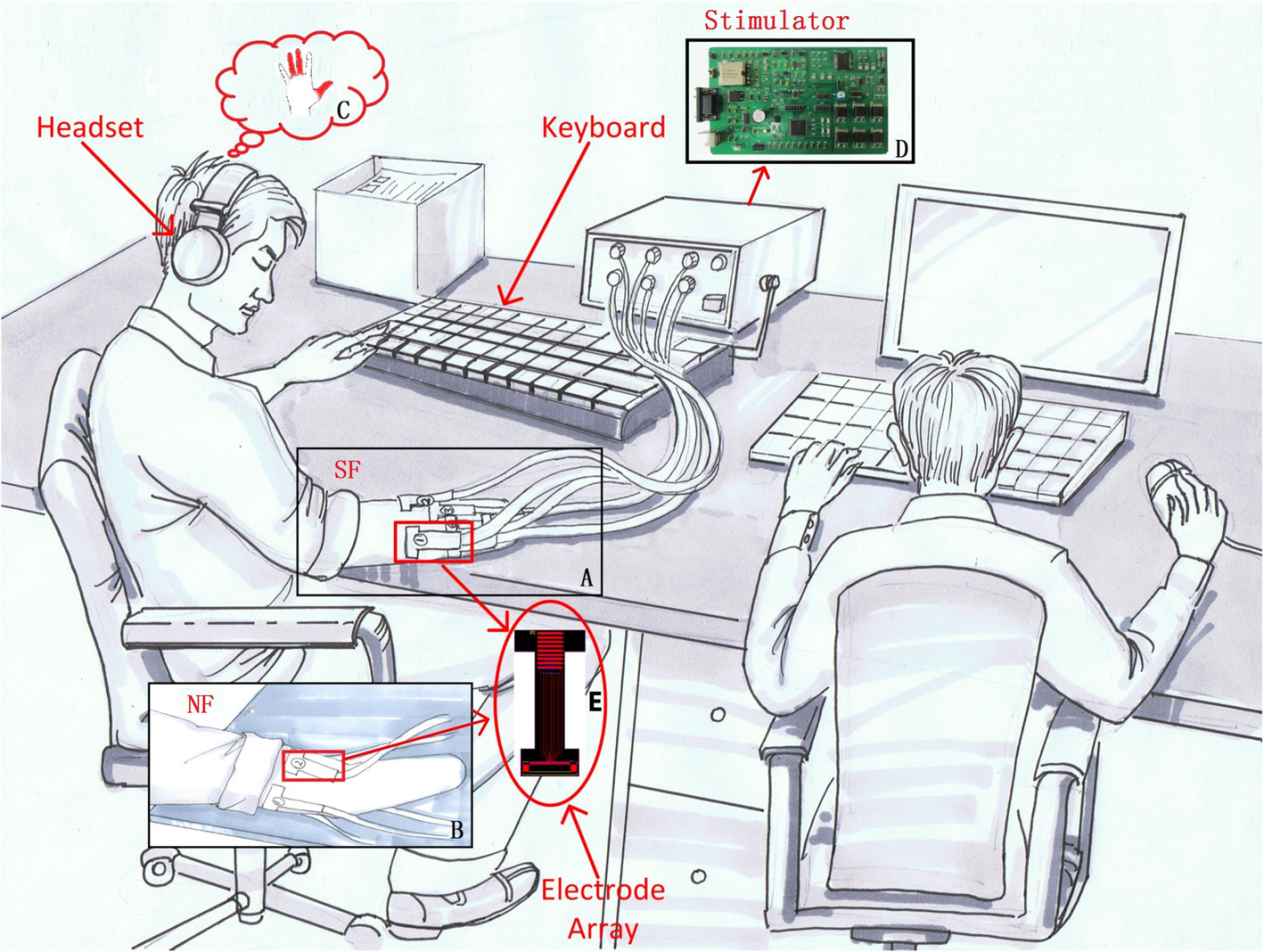
Experimental setup and structure.**A)** SF mode, **B)** NF mode, **C)** electrotactile sensation of the subject, **D)** custom-made electrical stimulator and **E)** electrode array.

The experimental setup is shown in Figure 1. The electrical stimulator (Figure 1-D) is comprised of a DC/DC boost converter module, a constant current source module and a full-bridge converter module. The output voltage can be up to 90V when the power supplies 12V voltage. The module of constant current source is converted from a constant voltage source. The range of output current is from 0 to 10 mA, and the direction can be either positive or negative. Four parameters of the current can be regulated via software: pulse amplitude, pulse width, pulse frequency, and pulse direction. The precision of amplitude, pulse width, and frequency is 0.01 mA, 10 *μ*s, 0.1 Hz, respectively.

The stimulation interface is a kind of custom-made electrode array with nine copper bars (Figure 1-E). To prevent chemical reactions during electrical stimulation and decrease the possibility of pricking sensations due to electric charge accumulation, the copper bars are covered with hydrogel. For the electrode array, the upper three copper bars are connected to the anode/cathode of the stimulator, the lower three copper bars are connected to the ground of the stimulator, and the middle three copper bars are not used in this work. All the copper bars do not share the same ground to prevent possible interference from the adjacent bars.

### Pilot study

A pilot test was conducted before the main experiment, and all subjects performed the following tasks.

#### Position selection of SF and NF

**Figure 2:**
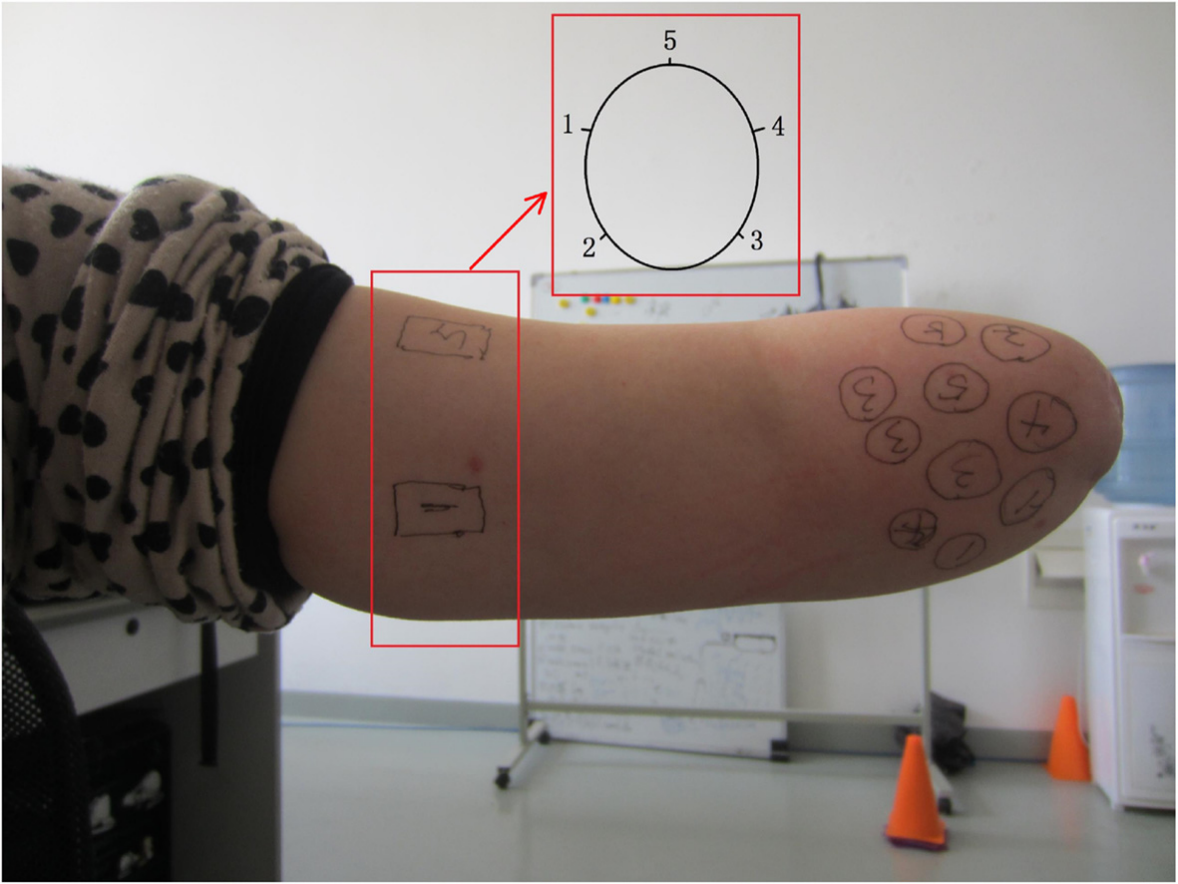
Stimulation positions for SF and NF. Circular marks on the stump are positions for SF, 1: phantom thumb; 2: phantom index finger; 3: phantom middle finger; 4: phantom ring finger; 5: phantom little finger. One phantom finger may have multiple positions. Rectangular marks on the upper arm are the stimulating positions for NF. The sequence of five NF positions is shown in the subfigure with cross-section view.

All subjects filled in a questionnaire reporting: dominant hand, cause of amputation, time of amputation, daily prosthesis usage/type and phantom limb pain by filling in the questionnaire. We evaluated phantom limb pain via a visual analogue scale (0 = minimum pain and 10 = maximum pain). All subjects had five phantom digits on their residual limb, and we located and marked specific positions of each phantom digit (see Figure 2) via direct touching and their own reports. The number 1−5 represented the phantom fingers from thumb to little finger. Please note that one phantom digit may have several positions due to uncontrollable reorganization of muscles and nerves in stumps after amputation. This process was for SF preparation.

The NF sites were chosen on the ipsilateral upper arm, not on the stump. Because all subjects had a somatotopical map on the stumps, the area left for NF was much smaller. Taking subject 3 as an example, her stump length was just 8 cm, and nearly all the area of her stump was occupied by the somatotopical map, so we could not find enough area for NF (5 channels). Even if enough area for NF could be found on the stump of some subjects, it was easy to mix with the SF area. Some subjects could not distinguish the exact boundaries clearly, thus the comparison between NF and SF would not be convincible in such case. In the end, the NF areas were selected on the upper arm for all subjects to maintain consistency. If NF was chosen on the stump for some subjects, while NF was chosen on the upper arm for other subjects, the comparison between NF and SF on different subjects would be not fair and just.

The well-known two-point discrimination test was adopted. First, it was performed on NF area of stumps and upper arms for the subjects. The results between stump and upper arm had were not different. This finding was similar to the results between forearm and upper arm reported in able-bodied subjects [30]. Therefore, it was reasonable to select NF on the upper arm in this work. Second, to select five positions on upper arm for delivering NF stimulation, we compared the sensation sensitivity between the longitudinal orientation and the transverse orientation on the seven subjects (see Table 2). The average results for discriminating two points showed that the subjects were more sensitive to distinguish positions along the transverse orientation, and it was in accordance with the phenomenon discovered by Perovic et al. [11]. This led to the conclusion that the positions for NF should be placed around the circumference of the arm as shown in Figure 2. Positions 1−5 indicated the five phantom digits.

**Table 2:** **Two-point discrimination assessment on NF**

**Subject**	**Longitudinal orientation**	**Transverse orientation**
	**(mm)**	**(mm)**
Subject 1	89	57
Subject 2	75	43
Subject 3	81	49
Subject 4	83	51
Subject 5	83	50
Subject 6	77	49
Subject 7	80	51
Average	81 ±4	50 ±4

#### Electrotactile stimulation

Stimulation current was set as monophasic and rectangular pulses. The amplitude of current was chosen to about 1.5mA, and was adjusted to assure the same perceived sensation to each subjects with the help of a psychophysical method (visual analog scale) [9]. For the pressure sensation, the stimulation current was negative, and we chose 100Hz as the stimulating frequency [31-33]. The subjects reported that the 100Hz-stimulation elicited a well-localized, continuous sensation resembling constant pressure on the surface of skin. Then we changed the pulse width from 0 to 500 *μ*s [34]. We recorded the minimum pulse width (*PW*_min_) for the subject when he reported the feeling of touch, and recorded the maximum pulse width (*PW*_max_) for the subject when he reported the feeling of pricking. For the vibration sensation, the current direction was positive. It was known that the strength of feeling was related to the velocity of vibration. Thus, we chose half of the maximum pulse width as the constant, and then changed the frequency from 1 to 75Hz (the current direction is positive) [31-33]. The maximum frequency (*f*_max_) was recorded for the subject when he reported that he/she couldn’t discriminate the interval of the pulses. This procedure was repeated three times in succession.

### Experimental protocol

Three evaluation indexes (position, type, and strength) were used to quantify the electrotactile sensation of the amputees. “Position” referred to amputees identifying which positions (see Figure 2) of skin were stimulated by the activated channels from stimulator. “Type” referred to amputees identifying the type of sensation (pressure or vibration). “Strength” referred to amputees identifying the strength of stimulation (weak, medium, or strong). For pressure sensation, we chose 20, 50, and 80 percent of the maximum pulse width (*PW*_max_) for stimulation, indicating three levels of strength. The current direction was negative and pulse frequency was 100Hz. For vibration sensation, we chose 20, 50, and 80 percent of the maximum frequency (*f*_max_) for stimulation, indicating three levels of strength. The current direction was negative and pulse width was half of the maximum pulse width (*PW*_max_). The finalized parameters of stimulation are shown in Table 3.

**Table 3:** **Setting of stimulating parameters**

**Feeling**	**Strength**	**Current**	**Frequency**	**Pulse width**
	Weak		20% *f*_max_	
Vibration	Medium	Positive	50% *f*_max_	50% *P* *W*_max_
	Strong		80% *f*_max_	
	Weak			20% *P* *W*_max_
Pressure	Medium	Negative	100 Hz	50% *P* *W*_max_
	Strong			80% *P* *W*_max_

Stimulation at different positions of SF or NF can elicit sensations of different phantom digits. In daily life, the thumb, index finger, and middle finger are used more frequently, while the ring finger and little figure are used less often. In order to reduce the experimental time for the amputees, not all combinations of five channels were considered. Instead, we chose one channel (1C), three channels (3C), and five channels (5C) for phantom digit sensations. For SF, 1C targeted the phantom thumb; 3C targeted the phantom thumb, index finger, and middle finger; 5C targeted all of the five phantom digits. For NF, the five positions were equally distributed around the circumference of the upper arm 6 cm above the elbow joint. 1C was in the middle of the glabrous area; 3C targeted three positions in the glabrous area; 5C targeted all the five positions on the circle of NF. Thus, there were six modalities of the stimulating channels for SF and NF, and we performed six experiments accordingly.

During each experiment, the subject wore a headset (Philips, Netherlands), which was used to receive a beep at the beginning of each trial (see Figure 1). Once the subject could identify position, type and strength of sensation for each stimulation, he pressed a control button of the keyboard by himself to stop the stimulation and make the computer record the trial’s response time. Then the subject verbally reported the answer to the experimenter. The experimenter input the subject’s answer to the computer and began the next trial. At the end of every experiment, the subject filled out a NASA-TLX questionnaire. The experimenter did not reveal the correct answer about the stimulation pattern to the subject in the experiments.

**Figure 3:**
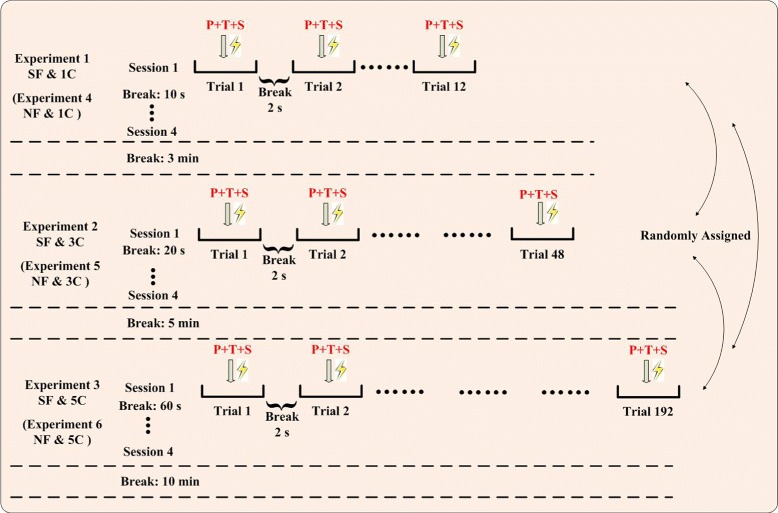
Protocol of electrotactile stimulation experiments. Three experiments (1C, 3C, 5C) were conducted each for SF and NF. All six experiments were assigned randomly. Each experiment had four sessions, and each session had some trials (12, 48, 192). There were breaks between trials, between sessions, and between experiments in order to reduce fatigue of subjects. P: position, T: type, S: strength.

The protocol of the six experiments is shown in Figure 3 and the order is random.

#### Experiment 1–1C for SF (1C/SF)

One-channel stimulation was performed. There were 2^1^ (the channel could be enabled or disabled) possible options for the evaluation index “position”, 2 possible options (pressure or vibration) for “type” and 3 possible options (weak, medium, or strong) for “strength”. Altogether, one session included 12 (2^1^∗2∗3=12) trials. There were four sessions, 48 trials (12∗4=48), and the overall duration lasted about 4 minutes.

#### Experiment 2–3C for SF (3C/SF)

Three-channel stimulation was performed. There were 2^3^ (each channel could be enabled or disabled) possible options for “position”, 2 possible options for “type” and 3 possible options for “strength”. Altogether, one session included 48 (2^3^∗2∗3=48) trials. There were four sessions, 192 trials (48∗4=192), and the overall duration of experiment 2 lasted about 18 minutes.

#### Experiment 3–5C for SF (5C/SF)

Five-channel stimulation was performed. There were 2^5^ (each channel could be enabled or disabled) possible options for “position”, 2 possible options for “type” and 3 possible options for “strength”. Altogether, one session included 192 (2^5^∗2∗3=192) trials. There were four sessions, 768 trials (192∗4=768), and the overall duration of experiment 3 was about 1–1.5 hours.

#### Experiments 4, 5, 6 (1C/NF, 3C/NF, 5C/NF)

Experiments 4, 5, 6 were for NF, which were similar to experiments 1, 2, 3 respectively. The only difference was that the stimulating positions were changed from the stump (SF) to the ipsilateral upper arm (NF).

### Self-Assessment Questionnaire

To evaluate the subjects’ mental workload during each experiment, we introduced the NASA-TLX questionnaire including six evaluated items: Mental Demands, Physical Demands, Temporal Demands, Own Performance, Effort, and Frustration ([2,35-37]). The questionnaire primarily included two parts: 1) subjects were required to compare two items in each pair of 15 pair-wise comparisons and chose the more important one; 2) subjects were required to rate each item from 0 to 100. Thus, we could get the weight of each item from part 1 (*W*_*i*_) and the score of each item from part 2 (*S*_*i*_). According to the following equation, we could calculate each subject’s mental workload index (*MWI*_*i*_) during each experiment. Higher mental workload index indicated more workload.

$$MW{I_{i}} = \frac{{\sum\limits_{i = 1}^{6} {{W_{i}} \cdot {S_{i}}} }}{{15}} $$

### Statistical analysis

Dependent variables were “accuracy” and “response time” except for NASA-TLX results. For NASA-TLX, the dependent variable was the NASA score (*MWI*). The term “accuracy” was defined as the ratio (percentage) between the number of correct trials and the number of total trials in the concerned situations, and it was calculated as

$$AC = \frac{number \; of \; correct \; trials}{{number \; of \; total \; trials}} \times 100\% $$

where the accuracies for Position, Type, Strength, and All were represented by *AC*_*p*_, *AC*_*t*_, *AC*_*s*_, *AC*_*a*_, respectively. Please note that in the condition “All”, the number of total trials in all three experiments (1C, 3C, 5C) is 12 ∗4 + 48 ∗ 4 + 192 ∗ 4=1008; the correct trials meant that the subjects should report all the three stimulating patterns correctly: position, type and strength, and the trials with any one pattern wrongly reported were not counted.

Four-way ANOVA was applied first, which had four factors: Feedback Site (SF, NF), Channel (1C, 3C, 5C), Type (pressure, vibration), Strength (low, medium, high). There were no other main-effect factors. Please note that “Position” indicated the positions of skin being stimulated, where the activated channels were applied. In other words, “Position” was a representation of the activated channels. Since channel was an effect already, “Position” was not considered any more. The “Subject” was a within-subjects factor, and the main effect of subject factor was not significant. It meant that successful accuracy and response time across experimental tasks were fully determined by the levels of the between-subjects factors, whereas the level of subject factor did not have a relevant influence. The results revealed that only the “Feedback Site (SF, NF)” and the “Channel (1C, 3C, 5C)” had interactions (*p*<0.05). Therefore, Type (pressure, vibration) and Strength (low, medium, high) were pooled across “Feedback Site” and “Channel”.

Since Feedback Site (SF, NF) and Channel (1C, 3C, 5C) had interactions, we used a simple-effect analysis to break down the ANOVA further into subsequent one-way ANOVA. The channel effects were analyzed for SF and NF, respectively. In SF, one-way ANOVA was used and the only factor was “Channel (1C, 3C, 5C)”, which was the same as that in NF. Feedback Site effects were analyzed for three channel conditions (1C, 3C, 5C), respectively, and one-way ANOVA was also used where the only factor was the “Feedback Site (SF, NF)”. Statistical significance was set to the level of *p*<0.05. We used the Bonferroni correction to do the post hoc pairwise comparisons. The *p*-values should be adjusted for multiple comparisons. For example, comparing the effects of different numbers of channels on SF and NF, the *p*-value should be adjusted to 0.05/3, and it was the same for the results of NASA-TLX.

## Results

### Comparison between SF and NF

**Figure 4:**
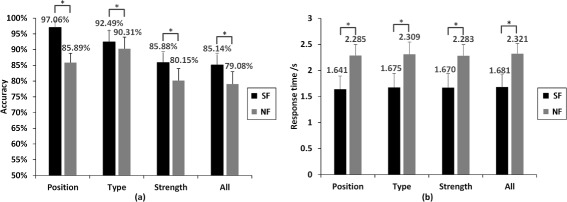
Average behavior results in comparison between SF or NF.**(a)** accuracy and **(b)** response time. Position: discriminating electrotactile position, and its accuracy is given by *A* *C*_*p*_. Type: discriminating the electrotactile type, and its accuracy is given by *A* *C*_*t*_. Strength: discriminating the electrotactile strength, and its accuracy is given by *A* *C*_*s*_. All: discriminating all the three electrotactile patterns, and its accuracy is given by *A* *C*_*a*_. The symbol ∗ between two bars * indicates statistical significance at the p=0.05 level. Error bars depict the standard deviation.

The averaged results of seven subjects are shown in Figure 4 with a comparative view between SF and NF. It was obvious that the accuracy and response time of SF were better than that of NF in general. If only SF was considered, the discrimination of position was the best (97.06%) and the discrimination of strength was the worst (85.88%). But if only NF was considered, the best was type discrimination (90.31%) and the worst was strength discrimination (80.15%). If SF was compared with NF in detail, we found that the largest difference was position discrimination (11.17%), and the least was type discrimination (2.18%). The average discrimination for SF was 6 percent higher than NF, and the average response time on SF was 600 ms faster than NF. There was a statistically significant difference (*p* < 0.001) between SF and NF for type and strength both accuracy and response time. If data of 1C were excluded, there was a statistically significant difference (*p* < 0.001) between SF and NF for position and all. In 1C modality, there was only one channel activated or deactivated, and the subjects need only judge if there was stimulation or not at a single position of skin. This was a very simple task, and no difference was observed between SF and NF.

### Comparison among different numbers of channels

**Figure 5:**
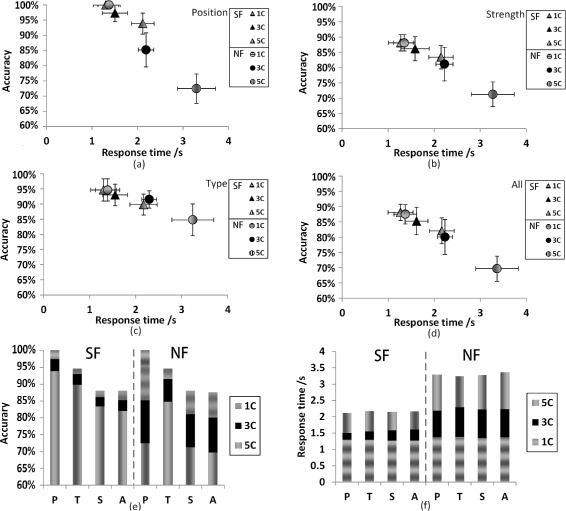
Average behavior results in comparison among three modalities (1C/3C/5C). In all the figures, 1C: one channel, 3C: three channels, 5C: five channels. In each of the four figures, for example, “SF, 1C” indicates that the result is achieved by SF through one channel, and “NF, 3C” indicates that the result is achieved by NF through three channels. The horizontal axis indicates the response time, and the perpendicular axis indicates the accuracy. Variances are represented by cross lines along with the triangles (SF) and circles (NF). **(e)** and **(f)** are the stacked plots of the same results of **(a)-(d)**. The left part of each figure is the performance of SF, and the right part belongs to the NF. In **(e)** and **(f)**, P: the performance of discriminating the position, and its accuracy is given by *A* *C*_*p*_ separately in 1C/3C/5C. T: the performance of discriminating the type, and its accuracy is given by *A* *C*_*t*_ separately in 1C/3C/5C. S: the performance of discriminating the strength, and its accuracy is given by *A* *C*_*s*_ separately in 1C/3C/5C. A: the performance of discriminating all of the three stimulation’s patterns, and its accuracy is given by *A* *C*_*a*_ separately in 1C/3C/5C. Error bars depict standard deviation.

Figure 5 shows the averaged results for discriminating position/type/strength in six experiments (SF: 1C/3C/5C, NF: 1C/3C/5C). The results were focused on effect of different numbers of stimulation channels. From (a)-(d) of the Figure 5, we got two interesting facts: 1) when only 1C was used, there was less difference in the discriminating accuracy and response time between SF and NF; 2) among the six experimental modalities, the performance for 1C of SF and NF was the best, since the subjects need only judge if there was stimulation or not. It was a very simple task, and the subjects nearly did not make any mistakes. The performance for 5C of NF was the worst, but the performance for 5C of SF was different from that for NF, even better than 3C of NF. From (e), (f) of the Figure 5, we noticed: when we chose SF, the difference of the performance between 5C and 3C was greater than that between 3C and 1C; 2) as the number of channels was increased, the deterioration of performance for NF was more severe than that for SF.

There were 15 pairwise comparisons on the performance of the six experiments. Within SF (1C, 3C, 5C), there were 3 pairwise comparisons, which was the same as that within NF (1C, 3C, 5C). There were 9 pairwise comparisons between SF and NF. The ANOVA results showed no statistically significant differences (*p* > 0.05) between 1C of SF and 3C of SF, 1C of SF and 1C of NF, 3C of SF and 1C of NF, 5C of SF and 3C of NF. Except for the above 4 pairs, all of the other 11 pairs were statistically different (*p* < 0.01).

### NASA-TLX Results

**Figure 6:**
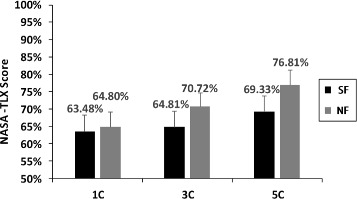
NASA-TLX mean scores of six experiments (SF/1C, SF/3C, SF/5C, NF/1C, NF/3C, NF/5C). 1C: one channel, 3C: three channels, 5C: five channels. Error bars depict standard deviation.

The mean NASA-TLX scores are shown in Figure 6. The 5C modality of NF required the highest mental workload (NASA-TLX: 76.81%). The 1C modality of SF required the lowest mental workload (NASA-TLX: 63.48%). The other two modalities with lower mental workload were 1C (NASA-TLX: 64.08%) and 3C (NASA-TLX: 64.81%). It seemed that the workload of NF was greater than that of SF in the same modality (the same number of stimulation channel). In order to analyze the statistical feature of all experimental NASA-TLX scores, we made a comparison between any two experiments. All of them were statistically different (*p* < 0.01), except the following four pairs: 1) 1C of SF and 3C of SF; 2) 1C of SF and 1C of NF; 3) 3C of SF and 1C of NF; 4) 3C of SF and 5C of NF. It was in accordance with the behavior results (discrimination accuracy and response time).

## Discussion

### General difference between SF and NF

Conventional theory of somatotopical mapping states that the sensitivity of touch sensation in different areas of the body is mainly due to the mechanoreceptor density in skin or the convergence effect of dorsal column nuclei (DCN) in the brainstem. The two-point discrimination assessment has shown that the forearm and upper arm of able-bodied subjects have similar tactile sensitivity [30]. However, the result of Figure 4 shows a large difference (11.17%) between SF (forearm) and NF (upper arm) on position discrimination, which cannot be explained by this conventional theory. The reason for the difference in position discrimination between SF and NF should be the effect of phantom digits. This means that some amputees feel their “original fingers” are touched when experimenters touch some specific parts of their stumps, and the tactile sensitivity of phantom fingers is better than arm.

Currently, some researchers have explained the phantom digit phenomenon in two ways [15,28,38]. First, with respect to peripheral nerve, the affected nerves that once deliver tactile information of fingers are attached to the tissue of the stump by suture during the amputation surgery. Some nerves may connect with the tactile receptors in the skin of stump. Thus, the tactile feedback channels have been reconstructed and amputees have phantom fingers. Second, with respect to the somatosensory cortex, the cortex that once represented the digits, hand or forearm region may be replaced by other parts of human body, such as the face, chest and stump. Thus, an amputee, whose cortex of the amputated hand is invaded by that of the stump, may also have phantom digits when his stump is touched.

For SF, when we deliver the tactile stimulation to the somatotopical positions on the subject’s amputated stump, it’s likely to stimulate the phantom digits. The ability of the amputees to discriminate positions is similar to that of the able-bodied. For NF, however, the stimulating positions are common places on the upper arm, which need subjects to set up the new mapping between the stimulating positions and specific fingers. It seems that the subjects should “learn” a technique that they are not familiar with, so they would feel confused when the stimulation channels are increased. Thus, the discrimination of position unquestionably deteriorates. That may be the cause of the different position’s discrimination between SF and NF.

The other performance of SF (type discrimination, strength discrimination, and response time) is also better than that of NF. The NASA-TLX questionnaire can provide some clues of the reason. We can see that the average NASA-TLX index by SF (65.87%) is less than NF (70.77%). This indicates that SF requires lower mental workload. On the contrary, it is harder and slower for the amputees to discriminate the stimulating modalities of NF.

### Influence of different numbers of channels

Behavior results among different numbers of channels are shown in Figure 5, and some interesting phenomena are discovered. For SF, since there is no statistically significant difference between 1C and 3C, it can be concluded that the ability of discriminating different stimulating modalities is nearly the same for the condition of fewer stimulation channels among the subjects. The cause of this surprising phenomenon may be that when one or three positions on the phantom digit mapping area are stimulated, it just looks like the corresponding real fingers (1 finger or 3 fingers) are stimulated. The mapping is strong and the mental burden is small, so the two stimulating modalities (1C and 3C) show similar results. But there is a statistically significant difference between 3C and 5C modalities. Despite the fact that 5C modality may also have a strong mapping of phantom digits, the distribution of current generated by five electrodes may interact, due to the small stimulation region. This may be a possible reason for the deteriorated performance on 5C of SF. There is further evidence from NASA-TLX index, which shows that 5C (69.33%) is greater than 3C (64.81%) (see Figure 6). This indicates that the 5C modality takes more mental workload. Thus, these two possible reasons might explain why the performance of the 5C modality was the worst.

For NF, there is a general deterioration in the accuracy and response time as the number of channels increases (*p* < 0.001). The more channels involved, the more attention is needed and this leads to more mistakes. Surprisingly, 1C of NF, 1C of SF, and 3C of SF are not statistically different. The reason for the similarity between 1C of NF and that of SF may be that the subjects only need to discriminate the type and strength of the stimulation without considering the stimulating position (there is only one stimulation channel). Thus, the discrimination ability on the type and strength between SF and NF does not make a difference.

Thus, if we have to seek a balance between good performance and rich sensation for electrotactile stimulation, some channels may be reduced leaving 3C/SF as the best choice.

### Pros and cons

This work does not aim to simply confirm that SF is superior to NF. We hope these results are helpful for the application of SF and NF. For some practical concerns about myoelectric controlled prosthesis, there are two problems: 1) some amputees do not have the phantom digit sensation, and we cannot provide the SF for amputees; 2) the area of stump is extremely valuable, and it can be a significant problem to arrange many electromyography (EMG) electrodes and electrical stimulation electrodes together.

Based on the results and discussions, we provide some feasible solutions to these practical problems (see Table 4) in three conditions. In condition 1 (#1), for amputees who have both the phantom digit sensation and large enough stump area, it is obvious to choose SF as the tactile feedback due to the better performance. Further, according to the discussion about the influence of different numbers of channels, we should choose 3C of SF to seek a balance between rich information (channels) and high performance. In condition 2 (#2), for amputees who have the phantom digit sensation, but do not have large enough stump area, we propose two solutions. 1) We can use 1C/NF instead of 1C/SF or 3C/SF when the demand of rich tactile information is not too high, and otherwise we use 3C/NF instead of 5C/SF. Because we found the performance of 1C/NF is similar to that of 1C/SF and 3C/SF, and the performance of 3C/NF is similar to that of 5C/SF in this study. 2) We can combine SF and NF together. For example, if only 1 channel can be placed on the stump, but we need 3 channels as the tactile feedback, we can combine 1C/SF and 2C/NF together. We think the performance of such a hybrid application will outperform that of 3C/NF. In condition 3 (#3), for amputees who do not have the phantom digit sensation, we can only use NF since the SF is invalid. We can make the choice just like the first solution of condition 2, i.e. 1C/NF or 3C/NF. Actually, NF performance is not different from SF if the number of channels is small.

**Table 4:** **Practical problems and solutions about tactile feedback for amputees**

**#**	**Phantom digit sensation**	**Area of stump**	**Solutions**
1	*√*	*√*	3C of SF
2	*√*	X	1C of NF, or 3C of NF,
			hybrid SF and NF
3	X	–	1C of NF, or 3C of NF

Specifically, when the demand of rich tactile information is high (the number of feedback channels must be 5 like five digits of a human hand) in condition 3, the only solution is to choose 5C/NF. Certainly, someone may question 5C/NF for the poor performance. However, our preliminary study has shown that reinforced learning (or training) for the amputees can improve the performance of NF. This indicates that the NF may show the similar performance as the SF, but it will require long training time and heavy mental workload. Future work will investigate challenges of reinforced learning and optimization for NF application.

## Conclusion

This work investigated multi-position, multi-type and multi-strength electrotactile feedback for amputees, towards closed-loop control of prosthesis in application. We focused on the difference between SF and NF, and the influence of different number of channels for tactile feedback. Through the study, we found that SF had better performance than NF in general. Furthermore, considering the subject’s behavior result and the task loading acquired from the NASA-TLX questionnaire, we recommend tactile feedback for three phantom digits as a good trade-off between better performance and more sensation information. Based on the results and analysis, we have provided possible solutions for solving practical problems about providing tactile feedback for amputees.

## References

[1] Childress DS (1980). Closed-loop control in prosthetic systems: historical perspective. Ann Biomed Eng..

[2] Gonzalez J, Soma H, Sekine M, Yu W (2012). Psycho-physiological assessment of a prosthetic hand sensory feedback system based on an auditory display: a preliminary study. J Neuroeng Rehabil.

[3] Herberts P, Körner L (1979). Ideas on sensory feedback in hand prostheses. Prosthet Orthot Int.

[4] Jiang N, Dosen S, Müller KR, Farina D (2012). Myoelectric control of artificial limbs–is there a need to change focus. IEEE Signal Process Mag.

[5] Altinsoy ME, Merchel S (2012). Electrotactile feedback for handheld devices with touch screen and simulation of roughness. IEEE Trans Haptics.

[6] Kaczmarek KA (2000). Electrotactile adaptation on the abdomen: Preliminary results. IEEE Trans Rehabil Eng.

[7] Kaczmarek KA, Tyler ME, Brisben AJ, Johnson KO (2000). The afferent neural response to electrotactile stimuli: preliminary results. IEEE Trans Rehabil Eng.

[8] Antfolk C, D’Alonzo M, Rosén B, Lundborg G, Sebelius F, Cipriani C. Sensory feedback in upper limb prosthetics. 2013. 10.1586/erd.12.68 23278223

[9] Buma DG, Buitenweg JR, Veltink PH (2007). Intermittent stimulation delays adaptation to electrocutaneous sensory feedback. IEEE Trans Neural Syst Rehabil Eng.

[10] Yamamoto A, Nagasawa S, Yamamoto H, Higuchi T (2006). Electrostatic tactile display with thin film slider and its application to tactile telepresentation systems. IEEE Trans Vis Comput Graph.

[11] Perović M, Stevanović M, Jevtić T, Štrbac M, Bijelić G, Vučetić Č, (2013). J Automatic Control.

[12] Antfolk C, Cipriani C, Carrozza MC, Balkenius C, Björkman A, Lundborg G (2013). Transfer of tactile input from an artificial hand to the forearm: experiments in amputees and able-bodied volunteers. Disabil Rehabil Assist Technol.

[13] Antfolk C, Bjorkman A, Frank SO, Sebelius F, Lundborg G, Rosen B (2012). Sensory feedback from a prosthetic hand based on air-mediated pressure from the hand to the forearm skin. J Rehabil Med.

[14] Cipriani C, Antfolk C, Balkenius C, Rosén B, Lundborg G, Carrozza MC (2009). A novel concept for a prosthetic hand with a bidirectional interface: a feasibility study. IEEE Trans Biomed Eng.

[15] Chai G, Li S, Sui X, Mei Z, He L, Zhong C, et al. Phantom finger perception evoked with transcutaneous electrical stimulation for sensory feedback of prosthetic hand. In: 2013 6th International IEEE/EMBS Conference on Neural Engineering (NER): 2013. p. 271–4.

[16] Antfolk C, D’Alonzo M, Controzzi M, Lundborg G, Rosen B, Sebelius F (2013). Artificial redirection of sensation from prosthetic fingers to the phantom hand map on transradial amputees: vibrotactile versus mechanotactile sensory feedback. IEEE Trans Neural Syst Rehabil Eng.

[17] Stepp CE, Matsuoka Y (2011). Object manipulation improvements due to single session training outweigh the differences among stimulation sites during vibrotactile feedback. IEEE Trans Neural Syst Rehabil Eng.

[18] Cipriani C, D’Alonzo M, Carrozza MC (2012). A miniature vibrotactile sensory substitution device for multifingered hand prosthetics. IEEE Trans Biomed Eng.

[19] Witteveen HJ, Droog EA, Rietman JS, Veltink PH (2012). Vibro-and electrotactile user feedback on hand opening for myoelectric forearm prostheses. IEEE Trans Biomed Eng.

[20] Damian DD, Arita A, Martinez H, Pfeifer R (2012). Slip speed feedback for grip force control. IEEE Trans Biomed Eng.

[21] Witteveen HJ, Luft F, Rietman JS, Veltink PH (2013). Stiffness feedback for myoelectric forearm prostheses using vibrotactile stimulation. IEEE Trans Neural Syst Rehabil Eng.

[22] Rombokas E, Stepp CE, Chang C, Malhotra M, Matsuoka Y (2013). Vibrotactile sensory substitution for electromyographic control of object manipulation. IEEE Trans Biomed Eng.

[23] D’Alonzo M, Cipriani C, Carrozza MC. Vibrotactile sensory substitution in multi-fingered hand prostheses: Evaluation studies. In: 2011 IEEE International Conference on Rehabilitation Robotics (ICORR): 2011. p. 1–6. 10.1109/ICORR.2011.5975477 22275675

[24] Antfolk C, Balkenius C, Lundborg G, Rosén B, Sebelius F (2010). Design and technical construction of a tactile display for sensory feedback in a hand prosthesis system. Biomed Eng Online.

[25] Saunders I, Vijayakumar S (2011). The role of feed-forward and feedback processes for closed-loop prosthesis control. J Neuroeng Rehabil.

[26] Seps M, Dermitzakis K, Hernandez-Arieta A. Study on lower back electrotactile stimulation characteristics for prosthetic sensory feedback. In: 2011 IEEE/RSJ International Conference on Intelligent Robots and Systems (IROS): 2011. p. 3454–459.

[27] Ehrsson HH, Rosén B, Stockselius A, Ragnö C, Köhler P, Lundborg G (2008). Upper limb amputees can be induced to experience a rubber hand as their own. Brain.

[28] Björkman A, Weibull A, Olsrud J, Henrik Ehrsson H, Rosén B, Björkman-Burtscher IM (2012). Phantom digit somatotopy: a functional magnetic resonance imaging study in forearm amputees. Eur J Neurosci.

[29] van der Riet D, Stopforth R, Bright G, Diegel O. Simultaneous vibrotactile feedback for multisensory upper limb prosthetics. In: IEEE Robotics and Mechatronics Conference (RobMech), 2013 6th: 2013. p. 64–9.

[30] Nolan MF (1982). Two-point discrimination assessment in the upper limb in young adult men and women. Phys Ther.

[31] Folgheraiter M, Gini G, Vercesi D (2008). A multi-modal haptic interface for virtual reality and robotics. J Intell Robot Syst.

[32] Kajimoto H, Kawakami N, Maeda T, Tachi S. Electro-tactile display with tactile primary color approach. Graduate School of Information and Technology, The University of Tokyo. 2004.

[33] Geng B, Yoshida K, Jensen W. Impacts of selected stimulation patterns on the perception threshold in electrocutaneous stimulation. J Neuroeng Rehabil. 2011; 8(9). 10.1186/1743-0003-8-9 PMC3045309 21306616

[34] Warren JP, Bobich LR, Santello M, Sweeney JD, Tillery SIH (2008). Receptive field characteristics under electrotactile stimulation of the fingertip. IEEE Trans Neural Syst Rehabil Eng.

[35] Hart SG. Nasa-task load index (nasa-tlx); 20 years later. In: Proceedings of the Human Factors and Ergonomics Society Annual Meeting: 2006. p. 904–8.

[36] Hart SG, Staveland LE (1988). Development of nasa-tlx (task load index): Results of empirical and theoretical research. Adv Psychol.

[37] Haapalainen E, Kim S, Forlizzi JF, Dey AK. Psycho-physiological measures for assessing cognitive load. In: Proceedings of the 12th ACM International Conference on Ubiquitous Computing: 2010. p. 301–10.

[38] Kuiken TA, Marasco PD, Lock BA, Harden RN, Dewald JP (2007). Redirection of cutaneous sensation from the hand to the chest skin of human amputees with targeted reinnervation. Proc Natl Acad Sci.

